# Prescribing, Medication Adherence, and Blood Pressure Goal Achievement Among Middle‐to‐Older Age Adults in a Primary Care Setting

**DOI:** 10.1155/ijhy/1613515

**Published:** 2026-11-06

**Authors:** Rehab Elhiny, Linda M. O’Keeffe, Tarun Das, Edel Burton, Seán R. Millar, Stephen Byrne, Maria Donovan, Margaret Bermingham

**Affiliations:** ^1^ Pharmaceutical Care Research Group School of Pharmacy University College Cork Cork Ireland ucc.ie; ^2^ Clinical Pharmacy Department Faculty of Pharmacy Minia University Minia Egypt minia.edu.eg; ^3^ School of Public Health University College Cork Cork Ireland ucc.ie; ^4^ MRC Integrative Epidemiology Unit at the University of Bristol University of Bristol Bristol UK bristol.ac.uk; ^5^ Population Health Sciences Bristol Medical School University of Bristol Bristol UK bristol.ac.uk; ^6^ School of Medicine University College Cork Cork Ireland ucc.ie; ^7^ Pharmacy Department Bon Secours Hospital Cork Ireland bonsecours.com

**Keywords:** antihypertensive prescription, blood pressure control, cardiovascular diseases risk factors, diastolic BP, hypertension, medication adherence, systolic BP

## Abstract

**Background:**

Blood pressure (BP) control among hypertensive people remains inadequate. This study aims to assess BP goal achievement among people prescribed antihypertensives, to examine medication nonadherence, and to identify demographics associated with goal achievements and medication nonadherence in this population.

**Methods:**

This was a cross‐sectional analysis of the Mitchelstown Cohort Study Rescreen data. Goal achievement was assessed in accordance with the 2013 European guidelines, the guidelines in place during data collection. A validated, self‐reported 3‐item medication nonadherence questionnaire was used, with a mean score ranging from 1 (Perfect Adherence) to 5 (Perfect Nonadherence). Nonadherence was defined as mean score > 1. Multiple imputation was used to impute missing data on covariates and outcomes. A sensitivity analysis was performed to assess the consistency of results by applying the 2024 European guideline recommendations. Multivariable logistic regression analysis was used to identify the factors associated with BP goal achievements and medication nonadherence.

**Results:**

Antihypertensives were prescribed to 567 participants. Based on imputed data, 63.8% of these achieved 2013 BP goal. Mean medication nonadherence score was 1.6 (SE ± 0.06), with 34.8% participants being nonadherent. Having multiple antihypertensives pills was associated with lower likelihood of achieving BP goals. In the sensitivity analysis, 38.4% achieved 2024 BP goal and in multivariable analysis, being overweight or obese was associated with not achieving the 2024 goals.

**Conclusion:**

The study highlights the gap between guideline BP goals and achieving these goals in real world practice. Key patient and medication factors were identified that could guide efforts to improve BP goal achievement.

**Trial Registration:** ClinicalTrials.gov identifier: NCT03191227

**Summary**
◦ Key points
• There is a gap in hypertension care between the recommended guideline blood pressure (BP) goals and achieving these goals in primary care, and this gap widens when stricter BP goals are applied.
• Agents acting on the renin–angiotensin–aldosterone system (RAAS) were the most prescribed antihypertensive agents and were more frequently prescribed than any other antihypertensive class to people who achieved their BP goals.
• Having a complex regimen of antihypertensive medications was associated with poor BP control in this population.
◦ Plain language summary:
• International guidelines set down BP goals for people with hypertension (high BP). However, achieving a healthy BP level among people with hypertension is a challenge for doctors internationally. Furthermore, some people do not take their BP‐lowering medication as it is prescribed to them and this may affect their BP levels. The aim of this study was to assess BP goal achievement among people prescribed BP‐lowering medications, to examine medication‐taking behavior, and to identify the patient factors associated with BP goal achievements among 567 people prescribed BP‐lowering medication in general practice. This study showed that 64% of participants achieved their BP goal. However, when a stricter BP goal was applied, just 38.4% achieved this. Most participants in this study took their BP‐lowering medications as prescribed. Patient factors that may make it harder to reach BP goal are being overweight or obese, having a complex treatment plan that includes multiple BP‐lowering medications and not taking BP‐lowering medications as prescribed. Women were more likely to take their BP‐lowering medications as prescribed than men. This study highlights the difficulty faced by people with high BP and their doctors in achieving BP goals.

## 1. Introduction

Cardiovascular diseases (CVDs) are a major cause of premature mortality and rising healthcare costs [1]. Hypertension is a key cause of CVDs including ischemic heart disease, stroke, and kidney disease and accounts for 8.5 million deaths worldwide annually [2]. Over the past 3 decades, the number of adults aged 30–79 years living with hypertension has risen from 650 million to 1.28 billion internationally, with half of these unaware of being hypertensive [2, 3].

Despite the availability and affordability of effective antihypertensive medication classes, several studies have reported that many people do not achieve adequate BP control as recommended in international hypertension management guidelines [4–6]. In a pooled analysis of data from 184 countries, BP control was 23% among women and 18% among men [2]. In Ireland, where an estimated one million adults aged 30–79 years had hypertension in 2019, 24% of people on treatment had a controlled BP [7]. A recent systematic review of Irish data reported suboptimal BP control, with 56% of people with hypertension achieving the recommended BP goal of < 140/90 mmHg [8]. Among people with diabetes, BP control ranged between 16.7% and 52.3% [8].

Globally, it is estimated that improving the rate of BP control to ≥ 50% would avert 76 million cardiovascular deaths by 2050 [7]. Certain patient factors may contribute to achieving this goal [3, 9]. Among these factors is the extent of patients’ adherence to prescribed antihypertensive treatment, which plays a significant role in BP control [3]. The World Health Organization defines adherence as “*the extent to which the person’s behavior (including medication taking) corresponds with the agreed recommendations from a healthcare provider*” [10, 11]. As described by the EMERGE guidelines, medication adherence includes medication initiation, implementation of the prescribed regime, and discontinuation of pharmacotherapy [10, 12]. Evidence has shown that medication nonadherence accounts for almost 75% of people who did not achieve BP goals [13–15]. The extent of medication nonadherence varies significantly between studies with different settings, designs, and methods and may influence reported nonadherence rates [16]. There are several reliable and valid tools for assessing medication nonadherence. These tools are classified into direct measures and indirect measures [11, 17]. Direct measures include directly observed therapy and urine analysis [11, 17]. Indirect measures include validated questionnaires and scales, many of which are self‐reported by patients or research participants [11, 17]. Among these questionnaires is the brief self‐reported 3‐item measure described by Voils et al. to assess the extent of medication nonadherence and validated in a hypertensive population [18–20].

While previous studies have highlighted the extent of medication nonadherence among people with hypertension, less is known about the demographic factors affecting medication nonadherence and BP goal achievements, especially in the Irish context. Factors identified as associated with BP control in tertiary settings include higher income, full health insurance, body mass index (BMI) in the normal range, nonuse of caffeinated drinks, having coronary heart disease, using nitrate medications, and having at least one annual visit to a subspecialist in a primary care setting [21, 22], while female sex, CVD risk measured by the Systematic COronary Risk Evaluation (SCORE), and clinical inertia were associated with poor BP control [23]. Irvin et al. reported that female sex, depression, and coronary artery disease were associated with poor medication adherence in a U.S. population aged ≥ 45 years with treatment‐resistant hypertension [15]. In addition, Kronish et al. found that among people prescribed antihypertensive medications, those prescribedangiotensin‐converting enzyme inhibitors (ACEIs) or angiotensin receptor blockers (ARBs) were twice as likely to be adherent compared to people prescribed diuretics or β‐blockers [24]. Thus, while medication nonadherence contributes to poor BP control, it does so through an interaction with patients’ clinical and demographic factors [25].

The primary aim of this study was to assess BP treatment goal achievement and medication nonadherence in a population of middle‐to‐older–aged people treated with antihypertensive medications in primary care. Secondary objectives were to identify factors associated with goal achievement and nonadherence and to describe the prescribing pattern of antihypertensive medications in relation to goal achievement.

## 2. Methods

The Mitchelstown Cohort Study recruited 2047 participants aged between 51 and 77 years at baseline in 2010 from a large general practice in the Cork region of Ireland. A follow‐up study was conducted in 2015, with 1378 of the baseline participants being rescreened [26]. The present study uses data from the 2015 rescreen only and is therefore a cross‐sectional analysis of the follow‐up data. The follow‐up involved two visits. The analysis in this study used data collected on the second visit. During the second visit, a computer‐assisted personal interview was used to collect various health information including medication adherence. In addition, physical examination was undertaken with measurements including weight, height, and resting BP. In this analysis, only participants who were prescribed antihypertensive medications were included. Detailed information on medical diagnosis and medication data was obtained during the annual screening of electronic health records which were recorded as part of routine general practice care. Full details regarding study design, sampling methods, and data collection were described previously in the cohort profile update [26].

Ethics committee approval was obtained from the Clinical Research Ethics Committee of University College Cork (The Cork and Kerry Diabetes and Heart Disease Study (Phase II) Mitchelstown Cohort). All participants gave signed informed consent, including permission to use their data for research purposes.

### 2.1. Demographic Data

Study participants provided data on age and sex. Sex was categorized into two groups: male and female. Age was categorized into four age ranges ≤ 59 years, 60–64 years, 65–69 years, and 70+ years. These categories were used to facilitate comparisons across age groups, while maintaining an adequate number of participants in each group for the statistical analysis and in line with previous analysis of this cohort [26].

The BMI was calculated by a healthcare professional using measurements of weight (kg) and height (m), which were taken during the physical examination. The weight and height of each participant were measured to the nearest 0.1 kg and 0.1 cm, respectively. Portable electronic Tanita WB‐100 MA weighing scales (Tanita Corporation, IL, USA) were placed on a firm, flat surface and were calibrated weekly to ensure accuracy. Height was measured using a portable Seca Leicester height/length stadiometer (Seca, Birmingham, United Kingdom). The BMI was calculated as weight in kg divided by the square of height [27]. The BMI was categorized into three categories: normal weight (18.5–24.9 kg/m^2^), overweight (25–29.9 kg/m^2^), and obese (≥ 30 kg/m^2^). Three participants categorized as underweight were excluded from the study to ensure stable model estimates in the multivariable analysis.

Education level, smoking status, and marital status were self‐reported as part of the computer‐assisted personal interview. Education level was included as a measure of socioeconomic status and was categorized into one of three categories: (i) primary level only (some primary/primary equivalent), (ii) secondary level (some secondary/secondary equivalent), or (iii) third level (diploma/primary university degree/postgraduate degree). Smoking status was categorized into current smoker, former smoker, and nonsmoker. Marital status was categorized into coupled (married or cohabitating) or single (separated, divorced, widowed, or single) [28].

### 2.2. Laboratory and Clinical Examination

Study participants’ BP was measured during the physical examination. Three independent measurements of systolic BP and diastolic BP were obtained with the subject in a seated position using an Omron M7 digital sphygmomanometer (Omron Healthcare Co. Ltd., Japan), and a mean of the three measures was calculated and used in this analysis [27, 29].

### 2.3. Medication Data and Diagnosis Data

Electronic health records were used to identify participants who were prescribed antihypertensive medications at the time of rescreen data collection. Medications were classified by their generic names and Anatomical Therapeutic Chemical (ATC) codes. Antihypertensive class ATC codes C02–C09 were identified. The treatment intensity was determined according to the number of prescribed antihypertensive medications and was categorized as (i) monotherapy, (ii) dual therapy, (iii) triple therapy, (iv) more than triple therapy. A further categorization of antihypertensive dual therapy and triple therapy was conducted to identify the regimen of the antihypertensive medications for those prescribed (i) free‐equivalent combinations (FECs), (ii) single pill combination (SPC), or (iii) both. This was examined because of the European Society of Cardiology (ESC) recommendation that suggests that SPC is associated with better adherence and persistence compared to FECs [30]. No polypharmacy was defined as taking less than or equal to four medications (including taking no medications). Polypharmacy was defined as the use of five to nine medications, and hyperpolypharmacy was defined as the use of ≥ 10 medications [31]. To minimize confounding by indication, people with previous diagnosis of heart failure, pulmonary embolism, atrial fibrillation, and anxiety were excluded to ensure that antihypertensive medications were not prescribed for any other indication.

### 2.4. Goal Achievement

The proportion of people who achieved target BP goal was evaluated, as set out in the 2013 European Society of Hypertension (ESH)/ESC guidelines for the management of arterial hypertension [32]. These guidelines were in use at the time of data collection. Systolic BP goal was < 140 mmHg in the entire population. The recommended diastolic BP goal was < 90 mmHg, except for people with diabetes, where the goal was < 85 mmHg [32].

### 2.5. Medication Adherence

Antihypertensive medication nonadherence was measured using the brief self‐reported three‐item measure described by Voils et al., a tool that is validated in a hypertensive population [20]. Participants rated the extent to which they took their medications or missed/skipped at least one dose or could not take any of their BP medications over the past 7 days. The questions were answered on a five‐point response scale of strongly disagree, disagree, neutral, agree, and strongly agree. Question 1 “*over the past 7 days, I took all doses of my BP medications*” was reverse‐scored with Strongly Disagree = 5 and Strongly Agree = 1; Question 2 “*over the past 7 days, I missed or skipped at least one dose of my BP medications*” and Question 3 “*over the past 7 days, I wasn’t able to take all of my BP medications*” were scored with Strongly Disagree = 1 and Strongly Agree = 5. A mean of the scores for the three questions was then calculated and ranged from 1 (Perfect Adherence) to 5 (Perfect Nonadherence) [20]. Following the approach described by Sagalla et al. participants were categorized as nonadherent if they had a mean score of > 1 [33].

### 2.6. Covariates

Covariates included in the analysis were age, sex, BMI, smoking status, education level, marital status, diabetes, statin therapy, regimen, polypharmacy, hyperpolypharmacy, and medication adherence.

### 2.7. Dealing With Missing Data

There were 320 participants who were prescribed an antihypertensive medication; had complete data for age, sex, systolic BP and diastolic BP, BMI, smoking status, and education level; and answered the medication nonadherence questionnaire. To increase efficiency and minimize selection bias, multivariate multiple imputation was used to impute missing data on covariates and outcomes in all participants with prescribed antihypertensive medications (*N* = 567). Twenty cycles of regression switching were performed and generated 20 imputation datasets using Stata Version 17.0 [34, 35]. The factors associated with goal achievements and medication adherence were examined using the multiple imputed datasets. Results were averaged across the results from each of these 20 datasets using Rubin’s rules, taking account of uncertainty in the imputation so that the standard errors for any regression coefficients (used to calculate 95% confidence intervals [CIs]) take account of uncertainty in the imputations and uncertainty in the estimate [34, 35]. To assume the missingness was missing at random, the imputed model included all the available demographic and clinical data. See Supporting Table 1 for the list of variables included in the multiple imputation models and how they were entered into the models.

### 2.8. Data Analysis

Statistical analysis using the imputed data was performed in Stata Version 17.0. The demographics of the study population were stratified by the achievement of BP goals. Continuous variables such as age, BMI, systolic BP, and diastolic BP are presented as mean and standard error (SE). Categorical variables were expressed as proportions.

Factors associated with achieving BP goals were examined using a multivariable logistic regression model. This model included age, sex, BMI, smoking status, education level, marital status, diabetes, statin therapy, regimen of antihypertensive medications, polypharmacy, hyperpolypharmacy, and medication adherence. Factors associated with medication adherence were then examined using a multivariable logistic regression model. This model included age, sex, BMI, smoking status, education level, marital status, diabetes, statin therapy, regimen of antihypertensive medications, polypharmacy, and hyperpolypharmacy. In the logistic regression models, odds ratios (ORs) and 95% CIs are shown.

Sensitivity analyses were conducted on the imputed and the observed dataset to check whether the results were consistent when applying the most recent European guideline recommendations. The proportion of people with a prescribed antihypertensive medication who achieved their target BP goals of 120–129/70–79 mmHg, as set out in the 2024 ESC Guidelines for the Management of Elevated Blood Pressure and Hypertension was assessed [36]. In addition, the factors associated with achieving these goals were examined using multivariable logistic regression analysis.

Multicollinearity among independent variables was assessed for the nonimputed dataset, with variance inflation factors (VIFs) calculated. A threshold of VIF above 2.5 was considered a potential multicollinearity.

The adjusted descriptive analysis was repeated in the observed dataset, among participants with complete cases on outcome and covariates (*N = 320*).

## 3. Results

Imputed data were available for 567 participants who were prescribed antihypertensive medications. The distribution of the observed data is broadly similar to the imputed datasets (Supporting Information Tables S2–S4). Mean age was 66.9 ± 0.22 (SE) years, 57.1% were males and 42.8% were females. Mean systolic BP was 133.1 ± 0.78 (SE) mmHg, and mean diastolic BP was 76.6 ± 0.4 (SE) mmHg; 13.7% had diabetes, and 8.2% were current smokers (Table 1). Among these participants, 362 (63.8%) achieved the 2013 ESC/ESH BP goals. Mean age and BMI were comparable between those who achieved BP goals and those who did not achieve BP goals (Table 1). The mean medication nonadherence score was 1.6 ± 0.06 (SE), with 198 (34.8%) participants considered nonadherent to their antihypertensive medications (Table S1). Among these, 80 (38.9%) nonadherent participants did not achieve the 2013 ESC/ESH goals.

**TABLE 1:** Patients’ characteristics based on goal achievements using the imputed data and the systolic BP and diastolic BP cutoff levels in the 2013 European Society of Cardiology/European Society of Hypertension guidelines.

Demographic characteristics	Achieved goals *N* = 362 (63.8%)	Did not achieve goals *N* = 205 (36.1%)	Total population (*N* = 567)
Age in years (mean, SE)	67.1 (0.28)	66.6 (0.35)	66.9 (0.22)
Body mass index (BMI) (kg/m^2^) (mean, SE)	29.7 (0.25)	30.8 (73.0)	30.1 (0.30)
Systolic BP (mmHg) (mean, SE)	122.2 (0.54)	152.3 (0.91)	133.1 (0.77)
Diastolic BP (mmHg) (mean, SE)	72.7 (0.40)	83.6 (0.75)	76.6 (0.43)
Diabetes (*n*, %)	48 (13.2)	30 (14.6)	78 (13.7)
Male (*n*, %)	195 (53.8)	129 (62.9)	324 (57.1)
Smoking status (*n*, %)			
Current smoker	26 (7.1)	21 (10.2)	47 (8.2)
Former smoker	146 (40.3)	85 (41.4)	231 (40.7)
Non smoker	190 (52.4)	99 (48.2)	289 (50.9)
Education level (*n*, %)			
Primary level	119 (32.7)	65 (31.5)	184 (32.3)
Second level	172 (47.4)	90 (43.9)	262 (46.1)
Third level	72 (19.7)	50 (24.5)	122 (21.4)
Marital status (*n*, %)			
Single	95 (26.2)	54 (26.3)	149 (26.2)
Couple	267 (73.7)	151 (73.6)	418 (73.7)
Polypharmacy status			
No polypharmacy	146 (40.6)	98 (47.8)	245 (43.2)
Polypharmacy	173 (47.7)	85 (41.4)	258 (45.5)
Hyperpolypharmacy	42 (11.6)	22 (10.7)	64 (11.2)

In multivariable regression analysis, having multiple antihypertensive pills (SPC or FEC) was associated with lower likelihood of achieving the 2013 goals compared with having one antihypertensive medication (OR 0.50; 95% CI 0.25, 0.97). Although not statistically significant, being obese (OR 0.60; 95% CI 0.32, 1.11) or being a current smoker (OR 0.55; 95% CI 0.28, 1.08) trended toward a negative association with achieving goals, whereas having prescribed statin therapy trended toward a positive association with achieving the 2013 BP goals (OR 1.04; 95% CI 0.93, 2.12) (Table 2). Female sex was associated with lower likelihood of nonadherence to antihypertensive medications compared to male sex (OR 0.59; 95% CI 0.37, 0.94) (Table 3).

**TABLE 2:** Multivariable logistic regression analysis of BP goal achievements using the imputed data and the cutoff levels set out in the 2013 European Society of Cardiology/European Society of Hypertension guidelines.

Associations with BP goal achievements 2013	Odds ratio	95% confidence interval	*p* value
Age categories: 65–69 years	Reference		
≤ 59 years	1.18	0.65, 2.13	0.572
60 – 64 years	1.08	0.66, 1.76	0.741
70+ years	1.17	0.75, 1.85	0.474

Sex: male	Reference		
Female	1.24	0.84, 1.82	0.266

Body mass index: normal weight	Reference		
Overweight	0.76	0.41, 1.40	0.384
Obese	0.60	0.32, 1.11	0.105

Smoke status: nonsmokers	Reference		
Current	0.55	0.28, 1.08	0.087
Former	0.88	0.59, 1.29	0.523

Education level: secondary level	Reference		
Primary level	0.99	0.64, 1.52	0.969
Third level	0.70	0.43, 1.12	0.144

Marital status: coupled	Reference		
Single	0.97	0.64, 1.46	0.893

No diabetes	Reference		
Diabetes	0.85	0.48, 1.49	0.580

No statin treatment	Reference		
Statin therapy	1.41	0.93, 2.12	0.097

Regimen: Monotherapy	Reference		
Free‐equivalent agents	0.86	0.54, 1.36	0.535
Single pill combination	1.24	0.69, 2.21	0.465
Free equivalent + SPC	0.50	0.25, 0.97	0.043

No polypharmacy	Reference		
Polypharmacy	1.35	0.68, 2.64	0.380
Hyperpolypharmacy	1.29	0.83, 1.99	0.245

Medication adherence: adherent	Reference		
Nonadherent	0.72	0.44, 1.17	0.189

*Note:* Mean VIF for the nonimputed dataset is 1.47.

**TABLE 3:** Multivariable logistic regression analysis of medication nonadherence among participants with prescribed antihypertensive medications using the imputed data.

Associations with nonadherence to antihypertensive medication	Odds ratio	95% confidence interval	*p* value
Age categories: 65–69 years	Reference		
≤ 59 years	1.06	0.50, 2.25	0.873
60 – 64 years	0.93	0.53, 1.63	0.808
70+ years	1.14	0.69, 1.89	0.601

Sex: male	Reference		
Female	0.59	0.37, 0.94	0.027

BMI: normal weight	Reference		
Overweight	0.85	0.40, 1.79	0.670
Obese	0.80	0.36, 1.75	0.575

Smoke status: nonsmokers	Reference		
Current	0.82	0.35, 1.90	0.647
Former	0.80	0.49, 1.29	0.367

Education level: secondary level	Reference		
Primary level	0.86	0.49, 1.49	0.598
Third level	0.73	0.41, 1.33	0.305

Marital status	Reference		
Single	1.20	0.72, 1.98	0.469

No diabetes	Reference		
Diabetes	0.80	0.38, 1.66	0.555

Regimen: monotherapy	Reference		
Free‐equivalent agents	0.66	0.36, 1.22	0.191
Single pill combination	1.30	0.66, 2.54	0.435
Free equivalent + SPC	0.54	0.22, 1.26	0.156

No polypharmacy	Reference		
Polypharmacy	1.00	0.57, 1.74	0.990
Hyperpolypharmacy	0.78	0.32, 1.88	0.585

No statin therapy	Reference		
Statin therapy	1.00	0.60, 1.67	0.976

*Note:* Mean VIF for the nonimputed dataset is 1.49.

In the sensitivity analysis, when the 2024 guideline was applied to this cohort, 38.4% achieved BP goals (Supporting Table 4). Having multiple antihypertensive pills (SPC or FEC) was associated with lower likelihood of achieving the 2024 goals compared with having one antihypertensive medication (OR 0.40; 95% CI 0.19, 0.84). Being overweight (OR 0.55; 95% CI 0.31, 0.97), obese (OR 0.50; 95% CI 0.28, 0.90), or nonadherent (OR 0.60; 95% CI 0.37, 0.96) was also associated with less likelihood of achieving these stricter goals. On the contrary, polypharmacy (OR 1.64; 95% CI 1.07, 2.54) and hyperpolypharmacy (OR 1.95; 95% CI 1.01; 3.79) were associated with higher likelihood of achieving 2024 BP goals (Table 4).

**TABLE 4:** Sensitivity analysis: multivariable logistic regression analysis of BP goal achievements using the imputed data and the systolic BP and diastolic BP cutoff levels in the 2024 European Society of Cardiology guidelines for the management of elevated blood pressure and hypertension.

Associations with BP goal achievements 2024	Odds ratio	95% confidence interval	*p* value
Age categories: 65–69 years	Reference		
≤ 59 years	0.80	0.43, 1.47	0.480
60–64 years	1.00	0.61, 1.63	0.955
70+ years	1.27	0.82, 1.99	0.277

Sex: male	Reference		
Female	0.76	0.52, 1.12	0.172

Body mass index: normal weight	Reference		
Overweight	0.55	0.31, 0.97	0.042
Obese	0.50	0.28, 0.90	0.021

Smoke status: nonsmokers	Reference		
Current	0.71	0.35, 1.44	0.355
Former	0.77	0.52, 1.13	0.193

Education level: secondary level	Reference		
Primary level	0.71	0.46, 1.10	0.127
Third level	0.65	0.39, 1.07	0.095

Marital status: coupled	Reference		
Single	1.02	0.67, 1.54	0.908

No diabetes	Reference		
Diabetes	0.71	0.40, 1.24	0.237

No statin treatment	Reference		
Statin therapy	1.49	0.98, 2.26	0.061

Regimen: monotherapy	Reference		
Free‐equivalent agents	0.89	0.56, 1.39	0.614
Single pill combination	1.13	0.64, 1.98	0.662
Free equivalent + SPC	0.40	0.19, 0.84	0.016

No polypharmacy	Reference		
Polypharmacy	1.64	1.07, 2.54	0.023
Hyperpolypharmacy	1.95	1.01, 3.79	0.046

Medication adherence: adherent	Reference		
Nonadherent	0.60	0.37, 0.96	0.036

*Note:* Mean VIF for the nonimputed dataset is 1.47.

The prescribing pattern of antihypertensive medications is outlined in Table 5. The most commonly prescribed antihypertensive classes were agents acting on the renin–angiotensin–aldosterone system (RAAS), prescribed to 71.0%, followed by β‐blockers (34.7%) and calcium‐channel blockers (20.6%). Two or more antihypertensives were prescribed to 45.8%, and FECs were the most prescribed antihypertensive combinations (25.2%). Among people who achieved their BP goals, RAAS agents and β‐blockers were prescribed more frequently compared to people who did not achieve their BP goals (72.0% vs 69.2%) and (37.2% vs 30.2%), respectively. Calcium‐channel blockers (28.2% vs 16.2%) were prescribed more frequently among people who did not achieve their BP goals than those who did achieve goal. Among those who achieved their BP goals, a higher proportion were prescribed two antihypertensives in a SPC compared to those who did not achieve their BP goals (13.2% vs 10.7%). However, those who did not achieve their BP goals were more likely to be prescribed 3 or more antihypertensives (17.1% vs 13.2%) and a more complex regimen of both FEC and SPC (10.7% vs 6.9%) compared to those who achieved their BP goals.

**TABLE 5:** Prescribing pattern of medications based on BP goal achievements using the imputed data and the systolic BP and diastolic BP cutoff levels set out in the 2013 European Society of Cardiology/European Society of Hypertension guidelines.

Medication data	Achieved goals *N* = 362 (63.8%)	Did not achieve goals *N* = 205 (36.1%)	Total population (*N* = 567)
Medication classes (*n*, %)			
Any renin–angiotensin–aldosterone system inhibitor	261 (72.0)	142 (69.2)	403 (71.0)
Any β‐blocker	135 (37.2)	62 (30.2)	192 (34.7)
Any calcium‐channel blocker	59 (16.2)	58 (28.2)	117 (20.6)
Any diuretic	3 (9.4)	18 (8.7)	21 (9.1)
Any alpha blocker	2 (0.5)	3 (1.5)	5 (0.8)
Intensity of antihypertensive medications (*n*,%)			
One antihypertensive	197 (54.4)	110 (53.6)	307 (54.1)
Two antihypertensives	117 (32.3)	60 (29.3)	177 (31.2)
Three or more antihypertensives	48 (13.2)	35 (17.1)	83 (14.6)
Classification of combination therapy (*n*, %)			
Free‐equivalent combination	92 (25.4)	51 (24.8)	143 (25.2)
Single pill combination	48 (13.2)	22 (10.7)	70 (12.3)
Both	25 (6.9)	22 (10.7)	47 (8.2)

## 4. Discussion

This study of middle‐ to older‐aged adults showed that 63.8% of participants with prescribed antihypertensive medications achieved the recommended 2013 BP goals; however, this declined to 38.4% when applying the stricter 2024 goals. Almost one‐third of the population self‐reported nonadherence to antihypertensive medications. Female sex was associated with lower risk of medication nonadherence, while having multiple antihypertensive pills prescribed was negatively associated with achievement of both 2013 and 2024 guideline BP goals. The prescribing patterns of antihypertensive medications differ among people who achieved goals and people who did not achieve goals. However, RAAS medications were the most commonly prescribed antihypertensive agents among participants in general.

High BP is a key preventable risk factor for CVDs worldwide [2, 37, 38]. Evidence has shown that the lower the BP targets, the greater the reduction in the risk of stroke and serious cardiovascular events [39]. However, BP control remains unsatisfactory worldwide. In 2017, the results from the May Measurement Month survey across 80 countries showed that 34.9% of respondents had hypertension; however, 46.3% of those on antihypertensive treatment did not reach the BP control of < 140/90 mmHg [37, 40]. The EURIKA study showed that across 12 European countries, just 38.8% had controlled BP, ranging from 32.1% in Greece to 47.5% in Turkey [23]. Similarly, in a nationwide survey in Italy, 85.8% of people with both diabetes and hypertension and 76.7% those with hypertension alone were prescribed antihypertensive medication, and only 25.4% and 30.8%, respectively had controlled BP [41]. While real world evidence of BP control using the recommended lower targets of systolic BP < 130 and diastolic BP < 80 remains scarce, the findings from the Akershus Cardiac Examination study of a cohort aged 62–65 years showed that only 23.0% of participants on antihypertensive medications achieved the 2024 ESC/ESH goals [36, 42].

In Ireland, evidence from the Irish Longitudinal Study on Aging showed that 63.7% of participants were considered hypertensive, with 58.9% of these receiving treatment. Of those who were on treatment, 51.6% were controlled [43]. The Irish Health Service Executive has implemented a Chronic Disease Management Programme in general practice since 2020. The program is targeted at specific chronic conditions including ischemic heart disease and Type 2 diabetes mellitus. Data from the first four years of the program showed that among 43,600 participants, 43.0% had SBP ≥ 140 mmHg and 44.5% had combined SBP ≥ 140 mmHg and DBP ≥ 90 mmHg [44]. These results are consistent with the findings presented here, demonstrating that BP control in Irish primary care settings remains suboptimal. Furthermore, previous analysis of Mitchelstown rescreen data has demomstrated thatLDL‐C control is likewise suboptimal [28], despite ongoing and focused efforts to target this risk factor.

Medication nonadherence is a clinical challenge in chronic diseases such as hypertension [3, 20]. While several methods have been developed to assess medication nonadherence, previous findings have shown that the extent of medication nonadherence varies considerably between studies with different settings, designs, and methods [15]. However, measures based on self‐reported medication nonadherence remain appealing because of their low cost and convenience [20]. In the current study, 34.8% of participants were considered nonadherent. A 2024 systematic review of Irish data showed that based on self‐reported medication adherence questionnaires, the rate of medication adherence among people with hypertension was between 27% and 89% [8]. Thus, the results presented here are situated in the range reported in the previous studies. Similarly, Voils et al. using the same 3‐item survey tool as used in this study reported a mean medication nonadherence score of 1.19 (±0.48) among 261 hypertensive participants, with a nonadherence rate of 36% among people prescribed antihypertensives on repeated, closely spaced survey administration [45]. A meta‐analysis of adherence to antihypertensive drugs found that the overall estimated medication nonadherence in 25 studies using the self‐reported 8‐item Morisky Medication Adherence Scale was 45.2% [14].

The findings of the current study suggest that males were more likely than females to be nonadherent to their antihypertensive medications. This aligns with previous literature that suggested the risk of nonadherence among males was 1.3 times higher than females [14]. Similarly, in France, among 2743 participants aged [3]55 years with prescribed antihypertensive medications, adherence was found to be higher among women compared to men (68.1% vs. 56.4%, *p* < 0.0001) [46]. Also, in keeping with our findings, the EURIKA study found prescription of statin therapy to be inversely associated with uncontrolled hypertension [23]. Although the EURIKA study attributed this association to the presence of confounding factors, a meta‐analysis of 45,723 participants from 49 placebo‐controlled randomized controlled trials showed that statin therapy significantly lowered both systolic BP (−1.42 mmHg) and diastolic BP (−0.82 mmHg) compared to those not prescribed statin therapy [47], although no mechanism was proposed for this effect. While being prescribed a SPC had no significant association with medication nonadherence in this study, a combination of FEC and SPC was negatively associated with achieving the 2013 and 2024 goals. Previous literature emphasized the role of SPC compared to FEC in reducing pill burden, thus improving medication adherence in addition to reducing therapeutic inertia [48, 49]. The present study showed a negative, abeit nonsignificant, association between smoking status and BP goal achievement. Gaya et al. showed that smoking cessation resulted in a reduction in systolic BP (−6 mmHg) and diastolic BP (−2 mmHg) among hypertensive former smokers after 12 weeks of treatment without any additional optimization of their antihypertensive treatment [50].

While clinical inertia cannot be examined in this analysis due to the nature of the cross‐sectional studies, the analysis of antihypertensive medicines showed that only 43% of the participants were prescribed antihypertensive medications as a SPC, which is broadly similar to the findings from the Irish Longitudinal Study on Aging, which reported 52.5% of hypertensive participants on combined antihypertensive medications during the same time period [43]. The 2013 ESH/ESC guidelines state that RAAS, diuretics, beta‐blockers, and calcium‐channel blockers are all suitable antihypertensive medications. They recommend initiating therapy with two antihypertensive medications. However, they favor SPC over multiple pills [30]. The results of the present study are consistent with the prescribing recommendations of the 2013 ESC/ESH guidelines as RAAS was the most commonly prescribed antihypertensive class. Interestingly, β‐blockers were the second most prescribed medication class, with 34.7% of participants prescribed a β‐blocker and 12.5% prescribed a β‐blocker as monotherapy. People prescribed an antihypertensive agent and with an overlapping indication such as heart failure or arrythmia were excluded from this analysis, meaning that hypertension was the indication for β‐blocker use in included participants. While this was consistent with the 2013 ESC/ESH guidelines, more recent European guidelines do not favor β‐blockers as first‐line therapy for people with hypertension, other than those with a compelling indication [30, 36, 51]. A higher proportion of people with three or more antihypertensive medications did not achieve BP goal, which may reflect greater treatment complexity in that group [32, 52].

### 4.1. Strengths and Limitations

This study provides valuable insights into prescribing of antihypertensive medications, achievement of BP goals, and the relationship between adherence and goal achievement in an Irish primary care setting. It is a cross‐sectional observational study. Therefore, BP was measured at a single timepoint only and pretreatment hypertensive levels are not available. Diagnosis data about hypertension were not available. Thus, it was not possible to identify people with untreated hypertension in this study. In accordance with previous studies, self‐reported medication nonadherence may underestimate nonadherence due to recall bias. The data were collected in a single primary care center in the greater Cork region, and this may limit the generalizability of the study. A key strength of the study is that multiple imputation was performed to address missing data and minimize the potential loss of statistical power associated with complete‐case analysis. In addition, we assessed the number of outcome events relative to the number of associations included in the regression model and confirmed that there were more than 10 outcome events per variable. This indicates that adequate statistical power was achieved throughout the analysis. Nevertheless, multiple imputation does not increase the underlying sample size or the amount of information contributed by the observed data. Furthermore, some categories within the independent variables had relatively small numbers of observations compared with other categories, which may have resulted in less precise estimates. However, the CIs remained reasonably narrow, including in the analyses based on the imputed datasets, suggesting adequately precise estimates. Finally, the additional analysis of goal achievements in accordance with the latest 2024 ESC goals improves the robustness of the results.

## 5. Conclusion

There is a gap between the guideline‐recommended BP goals and achieving these goals in clinical practice. Repeated assessment of medication adherence or use of more objective measures among people in primary care is needed to ensure reliable adherence estimates. Understanding the patient factors that are associated with BP goal achievements and medication adherence can facilitate and improve the care provided to people with hypertension in primary care.

## Funding

Dr. Rehab Elhiny is a recipient of a Graduate Studies Scholarship from the Cultural Affairs and Mission Department, Ministry of Higher Education and Scientific Research, Cairo, Egypt. Funding for this work was received from Novartis Ireland Ltd. The Mitchelstown Cohort Study was supported by a research grant to the HRB Centre for Health and Diet Research from the Irish Health Research Board (Reference HRC/2007/13).

## Disclosure

Prior postings and presentations: Data associated with this work were presented at the 53^rd^ European Society of Clinical Pharmacy Symposium, Grenoble, France, November 26–28, 2025, abstract available at Int J Clin Pharm (2026) 48:1069–1184, and at the European Society of Cardiology Preventive Cardiology Congress 2025, abstract available at Eur J Prev Cardiol 2025, 32, Suppl 1, i210.

## Ethics Statement

Ethics committee approval was obtained from the Clinical Research Ethics Committee of University College Cork (The Cork and Kerry Diabetes and Heart Disease Study (Phase II) Mitchelstown Cohort). All participants gave signed informed consent, including permission to use their data for research purposes.

## Conflicts of Interest

The authors declare no conflicts of interest.

## Supporting Information

Additional supporting information can be found online in the Supporting Information section.

## Supporting information


**Supporting Information** Table S1: Variables used in multivariable multiple imputation models. Table S2: Patients’ characteristics based on goal achievements using the imputed and observed data based on the systolic BP and diastolic BP cutoff levels in the 2013 ESC/ESH guidelines goals. Table S3: Patients’ characteristics based on BP goal achievements using the imputed data and observed data based on the systolic BP and diastolic BP cutoff levels in the 2024 ESC Guidelines for the management of elevated BP and hypertension. Table S4: Medication nonadherence among participants using the imputed data and observed data.

## Data Availability

The data that support the findings of this study are available on request from the corresponding author.
