# Supplementary material for: Prescribing, Medication Adherence, and Blood Pressure Goal Achievement Among Middle‐to‐Older Age Adults in a Primary Care Setting
Source: Int J Hypertens. 2026 Nov 6;2026:1613515. doi: 10.1155/ijhy/1613515 (PMC13743861; doi:10.1155/ijhy/1613515)
Supplement: Supplementary file 1 [file IJHY-2026-1613515-s001.docx]

**Prescribing, medication adherence and blood pressure goal achievement among middle-to- older age adults in a primary care setting**

Rehab Elhiny ^1,2^, Linda M O’Keeffe ^3,4,5^, Tarun Das^6^, Edel Burton ^1,7^, Seán R. Millar ^3^, Stephen Byrne ^1,2^, Maria Donovan ^1^, Margaret Bermingham ^1^

**Additional file:**

**Table S1:** Variables used in multivariable multiple imputation models.

**Table S2:** Patients’ characteristics based on goal achievements using the imputed and observed data based on the systolic BP and diastolic BP cut off level in the 2013 European Society of Cardiology (ESC)/ European Society of Hypertension guidelines (ESH) goals.

**Table S3**: Patients’ characteristics based on BP goal achievements using the imputed data and observed data based on the systolic BP and diastolic BP cut off level in the 2024 ESC Guidelines for the management of elevated blood pressure and hypertension.

**Table S4:** Medication non-adherence among participants using the imputed data and observed data.

Supplementary Table 1. Variables used in multivariable multiple imputation models

| **Variable** | **Type of variable** | **Model used to predict missing data** |
| --- | --- | --- |
| Marital status | Categorical (2 categories) | n/a, complete cases |
| Sex | Categorical (2 categories) | n/a, complete cases |
| Age categories | Categorical (4 categories) | n/a, complete cases |
| Systolic BP | Continuous | n/a, complete cases |
| Diastolic BP | Continuous | n/a, complete cases |
| Smoking status | Categorical (3 categories) | n/a, complete cases |
| Body mass index categories | Categorical (3 categories) | n/a, complete cases |
| Diabetes | Categorical (2 categories) | n/a, complete cases |
| Polypharmacy | Categorical (3 categories) | n/a, complete cases |
| Statin therapy | Categorical (2 categories) | n/a, complete cases |
| Education level | Categorical (3 categories) | Ordinal logistic regression |
| Number of antihypertensives | Categorical (3 categories) | n/a, complete cases |
| Medication classes | Categorical (4 categories) | n/a, complete cases |
| Question 1 | Ordered categories (5 categories) | Ordinal logistic regression |
| Question 2 | Ordered categories (5 categories) | Ordinal logistic regression |
| Question 3 | Ordered categories (5 categories) | Ordinal logistic regression |
| Average score | Continuous | Passive imputation |
| Medication non-adherence | Categorical (2 Categories) | Passive imputation |

Supplementary Table 2. Patients’ characteristics based on goal achievements using the imputed and observed data based on the systolic BP and diastolic BP cut off level in the 2013 European Society of Cardiology (ESC)/ European Society of Hypertension guidelines (ESH) goals^1^.

| Variables | % of the imputed data | Achieved goals | | Didn’t achieve goals | |
| --- | --- | --- | --- | --- | --- |
|  |  | Imputed data  (N = 362)  (63.8%) | Observed data  (N = 217)  (67.8%)  (without imputation) | Imputed  (N = 205)  (36.2%) | Observed  (N = 103)  (32.2%)  (without imputation) |
| Age in years |  | 67.1 (0.28) | 67.3 (0.37) | 66.6 (0.35) | 66.4 (0.53) |
| Body mass index (BMI) (Kg/m^2^ ) |  | 29.7 (0.25) | 29.7 (0.3) | 30.8 (0.73) | 31.4 (1.4) |
| Systolic BP (mmHg) |  | 122.2 (0.54) | 122.0 (0.67) | 152.4 (0.92) | 152.4 (1.29) |
| Diastolic BP (mmHg) |  | 72.7 (0.40) | 73.1 (0.53) | 83.6 (0.75) | 83.1 (1.03) |
| Diabetes |  | 13.2 | 13.3 | 14.6 | 10.6 |
| Male |  | 53.8 | 52.5 | 62.9 | 61.1 |
| Smoking status (n,%) | | | | | |
| Current smoker |  | 7.2 | 5.1 | 10.2 | 9.7 |
| Former smoker |  | 40.3 | 43.3 | 41.5 | 42.7 |
| Non-smoker |  | 52.4 | 51.6 | 48.2 | 47.5 |
| Marital status (n,%) | | | | | |
| Single |  | 26.2 | 22.5 | 26.3 | 26.2 |
| Coupled |  | 73.7 | 77.4 | 73.6 | 73.7 |
| Education level (n,%) | | | | | |
| Primary level | 7.7% | 32.3 | 32.7 | 31.6 | 29.1 |
| Second level |  | 47.8 | 47.0 | 44.0 | 45.6 |
| Third level |  | 19.7 | 20.3 | 24.4 | 25.2 |
| Antihypertensive intensities (n,%) | | | |  |  |
| Monotherapy |  | 54.4 | 50.6 | 53.6 | 43.6 |
| Dual therapy |  | 32.3 | 33.2 | 29.2 | 35.9 |
| Triple therapy |  | 11.6 | 14.3 | 13.2 | 18.4 |
| More than triple therapy |  | 1.6 | 1.8 | 3.9 | 1.9 |
| Antihypertensive regimen (n,%) | | | |  |  |
| Free equivalent combination |  | 55.7 | 56.1 | 53.6 | 62.1 |
| Single pill combination (SPC) |  | 29.0 | 28.0 | 23.1 | 22.4 |
| Free equivalent + SPC |  | 15.1 | 15.8 | 23.1 | 15.5 |
| Antihypertensive Class |  |  | | | |
| Renin angiotensin aldosterone system inhibitor |  | 72.1 | 80.6 | 69.2 | 72.8 |
| β blockers |  | 37.2 | 33.2 | 30.2 | 27.2 |
| Calcium channel blocker |  | 16.2 | 16.5 | 28.2 | 31.1 |
| Diuretic |  | 9.4 | 9.6 | 8.7 | 11.6 |
| Alpha blockers |  | 0.5 | 0.46 | 1.4 | 1.94 |
| Medication adherence (n,%) | | | |  |  |
| Adherent |  | 67.4 | 76.0 | 61.1 | 70.8 |
| Non-adherent |  | 32.5 | 23.9 | 38.9 | 29.1 |

Supplementary Table 3. Patients’ characteristics based on BP goal achievements using the imputed data and observed data based on the systolic BP and diastolic BP cut off level in the 2024 ESC Guidelines for the management of elevated blood pressure and hypertension^2^.

|  | **Achieved goals** | | **Did not achieve goals** | |
| --- | --- | --- | --- | --- |
|  | **Imputed data**  **(N = 218)** | **Observed data**  **(N = 130)** | **Imputed data**  **(N = 349)** | **Observed data**  **(N = 190)** |
| Age in years  (mean, SE) | 67.5 (0.35) | 68.2 (0.44) | 66.5 (0.28) | 66.2 (0.40) |
| Body mass index (BMI) (kg/m^2^) (mean, SE) | 29.6 (0.34) | 29.9 (0.39) | 30.4 (0.45) | 30.5 (0.77) |
| Systolic BP (mmHg) (mean, SE) | 116.6 (0.55) | 116.8 (0.70) | 143.4 (0.81) | 142.0 (1.12) |
| Diastolic BP (mmHg) (mean, SE) | 70.0 (0.42) | 81.2 (0.53) | 70.1 (0.55) | 80.9 (0.69) |
| Diabetes (n,%) | 13.7 | 13.8 | 13.7 | 11.5 |
| Male (n,%) | 56.8 | 56.2 | 57.3 | 54.7 |
| Smoke status (n,%) | | | | |
| Current smoker | 7.7 | 4.6 | 8.6 | 7.9 |
| Former smoker | 39.4 | 43.1 | 41.5 | 43.2 |
| Non smoker | 52.7 | 52.3 | 49.9 | 48.9 |
| Education level (n,%) | | | | |
| Primary level | 31.2 | 33.1 | 33.0 | 30.5 |
| Second level | 50.3 | 48.5 | 43.6 | 45.3 |
| Third level | 18.5 | 18.5 | 23.3 | 24.2 |
| Marital status (n,%) | | | | |
| Single | 26.6 | 25.4 | 26.0 | 22.6 |
| Coupled | 73.9 | 74.6 | 73.9 | 77.4 |
| Medication adherence (n,%) | | | | |
| Adherent | 61.7 | 80.0 | 70.6 | 70.5 |
| Non adherent | 38.3 | 20.0 | 29.3 | 29.5 |

Supplementary Table 4. Medication non-adherence among participants using the imputed data and observed data.

|  |  |  | Imputed data | | | | | Observed data | | | | |
| --- | --- | --- | --- | --- | --- | --- | --- | --- | --- | --- | --- | --- |
| Item | Adherence statement | % of miss data | Strongly  disagree | Disagree | Neutral | Agree | Strongly  Agree | Strongly  disagree | Disagree | Neutral | Agree | Strongly  Agree |
| 1 | Over the past 7 days, I took all doses of my BP medications | 29.8 | 56  (9.8) | 8  (1.6) | 3  (0.5) | 28 (4.9) | 472  (83.3) | 25  (7.8) | 4 (1.3) | 2 (0.6) | 11 (3.4) | 278 (86.8) |
| 2 | Over the past 7 days, I missed or skipped at least one dose of my BP medications | 35.6 | 428  (75.5) | 21  (3.7) | 16  (2.8) | 14  (2.4) | 88  (15.6) | 266  (83.1) | 7 (2.2) | 6 (1.9) | 6 (1.9) | 35 (10.9) |
| 3 | Over the past 7 days, I wasn’t able to take all of my BP medications | 36.6 | 471  (83.0) | 10  (1.7) | 6  (1.0) | 5  (1.0) | 75  (13.3) | 282 (88.1) | 3 (0.9) | 2 (0.6) | 1 (0.3) | 32 (10.0) |

Reference:

1.	Authors/Task Force Members, Mancia G, Fagard R, et al. 2013 ESH/ESC Guidelines for the management of arterial hypertension: The Task Force for the management of arterial hypertension of the European Society of Hypertension (ESH) and of the European Society of Cardiology (ESC). *Eur Heart J*. 2013;34(28):2159-2219. doi:10.1093/eurheartj/eht151

2.	McEvoy JW, McCarthy CP, Bruno RM, et al. 2024 ESC Guidelines for the management of elevated blood pressure and hypertension: Developed by the task force on the management of elevated blood pressure and hypertension of the European Society of Cardiology (ESC) and endorsed by the European Society of Endocrinology (ESE) and the European Stroke Organisation (ESO). *Eur Heart J*. 2024;45(38):3912-4018. doi:10.1093/eurheartj/ehae178
